# TENT-5 polyadenylates and regulates male-specific transcripts in *Caenorhabditis elegans*

**DOI:** 10.1093/g3journal/jkag058

**Published:** 2026-03-23

**Authors:** Zuzanna Mackiewicz, Vladyslava Liudkovska, Andrzej Dziembowski

**Affiliations:** Laboratory of RNA Biology, International Institute of Molecular and Cell Biology, Trojdena 4, Warsaw 02-109, Mazovia, Poland; Laboratory of Stem Cell RNA Metabolism, IMol Polish Academy of Sciences, Flisa 6, Warsaw 02-247, Mazovia, Poland; Laboratory of RNA Biology, International Institute of Molecular and Cell Biology, Trojdena 4, Warsaw 02-109, Mazovia, Poland

**Keywords:** sexual dimorphism, poly(A) tail metabolism, TENT-5 poly(A) polymerase, male-specific genes, *C. elegans* male physiology, seminal fluid components, vas deferens, Animalia

## Abstract

Polyadenylation is an important post-transcriptional process that governs mRNA stability and expression. Advancements in direct RNA sequencing in recent years have clarified many aspects of this intricate regulation, revealing the influence of various factors. Here, we used Nanopore Direct RNA Sequencing to investigate the association between genome-wide mRNA poly(A) tail profiles and sexual dimorphism in *Caenorhabditis elegans*. Our results demonstrate sex-dependent differences in both gene expression and poly(A) tail metabolism. Notably, we discovered that cytoplasmic poly(A) polymerase terminal nucleotidyltransferase family of proteins (TENT-5) polyadenylates multiple male-specific transcripts, predominantly encoding putative seminal fluid components with predicted extracellular localization. TENT-5 expression in a male-specific tissue—vas deferens, corroborates its functional significance. Intriguingly, despite extensive TENT-5-mediated polyadenylation of male-specific transcripts, males devoid of TENT-5 show no detectable abnormalities in mating behavior, spermatid morphology, or fertility. Our findings suggest that TENT-5 might play a role in regulating sex-related processes in males, although the physiological consequences remain to be fully elucidated.

## Graphical Abstract

**Graphical Abstract:**
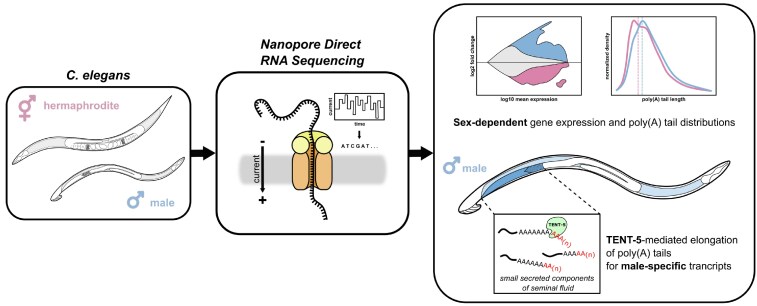

## Introduction

Polyadenylation is a crucial post-transcriptional process that modifies RNA species by adding adenosines to their 3′ ends, thereby influencing RNA stability and function within the cell. This modification primarily occurs in the nucleus, catalyzed by canonical poly(A) polymerase, and is essential for mRNA biogenesis and subsequent export to the cytoplasm (Jalkanen et al. 2014; Passmore and Coller 2022). Upon reaching the cytoplasm, mRNAs associate with poly(A) binding proteins, which are critical for maintaining mRNA stability and enabling efficient protein synthesis (Wigington et al. 2014; Passmore and Coller 2022). During the later lifespan of mRNA, poly(A) tails undergo further modifications. Deadenylating enzymes, such as CCR4-NOT or PAN2/3, gradually shorten poly(A) tails, directing mRNAs to degradation (Zhang et al. 2010; Yan 2014; Passmore and Coller 2022). However, poly(A) tails can also be re-adenylated in the cytoplasm by noncanonical poly(A) polymerases (ncPAPs), which are members of the terminal nucleotidyltransferase family of proteins (TENTs) (Yu and Kim 2020; Liudkovska and Dziembowski 2021). Cytoplasmic polyadenylation enhances stability, promotes the expression of selected transcripts, and prevents their degradation, contributing to the complex dynamics of poly(A) tails and regulation of gene expression (Yu and Kim 2020; Liudkovska and Dziembowski 2021).

Recent advances in RNA sequencing technologies have enabled more comprehensive studies of poly(A) tail metabolism (Brouze et al. 2023). Genome-wide poly(A) tail profiling has been carried out for various organisms, including yeast (Subtelny et al. 2014 ; Tudek et al. 2021), nematodes (Lima et al. 2017; Legnini et al. 2019; Liudkovska et al. 2022), mice (Subtelny et al. 2014; Bilska et al. 2020; Begik et al. 2023; Brouze et al. 2024), and humans (Chang et al. 2014; Subtelny et al. 2014; Legnini et al. 2019). These studies revealed organism-specific characteristics of poly(A) tails and their dependence on multiple factors such as developmental stage, age, tissue localization, and response to environmental cues. While poly(A) tail length is often positively associated with mRNA half-life and translation efficiency, this relationship is not always consistent. During early embryogenesis, longer poly(A) tails enhance mRNA stability and translation (Subtelny et al. 2014; Lim et al. 2016; Passmore and Coller 2022). However, in somatic cells, poly(A) tail length often shows a poor or even negative correlation with mRNA expression levels and translational activity (Subtelny et al. 2014; Lima et al. 2017; Legnini et al. 2019). For instance, mRNAs encoding highly abundant housekeeping machinery, such as ribosomal proteins, typically have short tails. Conversely, longer tails are predominantly found on lowly expressed mRNAs, which often encode regulatory proteins (Castellano and Bazzini 2017; Lima et al. 2017). Moreover, poly(A) tail dynamics is affected by additional layers of regulatory control, including poly(A)-modifying enzymes that influence numerous physiological processes. For example, cytoplasmic polyadenylation by GLD2 (Germ Line Development 2)/TENT2 is indispensable for proper germline development and fertility in nematodes (Schmid et al. 2009; Nousch et al. 2017; Boag et al. 2018) and flies (Benoit et al. 2008; Sartain et al. 2011). In mammals, a similar role is observed for the recently discovered cytoplasmic TENT5 ncPAPs (Brouze et al. 2024). Additionally, members of the TENT5 family have been implicated in regulating humoral and innate immune responses by stabilizing mRNAs encoding immunoglobulins and secreted defense proteins (Bilska et al. 2020; Liudkovska et al. 2022) or even re-adenylation and stabilization of mRNA vaccines (Krawczyk et al. 2025). Despite these findings, the high complexity of cytoplasmic polyadenylation requires further examination to elucidate the interplay between poly(A)-modifying enzymes, the mechanisms driving their substrate specificity, and their effects on various groups of transcripts during multiple physiological processes and conditions (Liudkovska and Dziembowski 2021).

Here, we demonstrate that sexual dimorphism is a critical factor that strongly influences polyadenylation profiles. For our studies, we used *Caenorhabditis elegans*, which features two distinct sexes: self-fertile hermaphrodites (XX) and males (X0), comprising approximately 0.05% of the population. Utilizing Oxford Nanopore Direct RNA Sequencing (DRS), a PCR-free approach that sequences native RNA and allows direct estimation of poly(A) tails (Brouze et al. 2023 ), we uncovered profound differences in gene expression and poly(A) length distributions between hermaphrodites and males. Notably, we showed that cytoplasmic TENT-5 ncPAP is responsible for the regulation of male-specific transcripts that mainly encode secreted seminal fluid components. Our work provides new and interesting insights into the polyadenylation process, expanding our knowledge of both TENT-5 functions and *C. elegans* male physiology and genetics.

## Materials and methods

### *C. elegans* culture and growth conditions

The following *C. elegans* strains were used: wild type N2 Bristol, BS553 (*fog-2(oz40) V*), and DR94 (*unc-45(m94) III*), obtained from the Caenorhabditis Genetics Center (CGC); *tent-5(tm3504) I*, obtained from the National Bioresource Project of Japan (NBRP); and ADZ21 (*tent-5(rtt6[tent-5::gfp::3xflag]) I*), previously generated in our laboratory (Liudkovska et al. 2022). All strains were cultured at 20 °C on nematode growth medium (NGM) plates seeded with *E. coli* HB101 as a food source. Male enrichment of N2, *tent-5(tm3504)*, and *tent-5(rtt6) C. elegans* cultures was achieved through mating, with one hermaphrodite and five males of the same genotype placed on plates with a drop of *E. coli* HB101. Successful mating resulted in up to 50% male enrichment.

### Preparation of samples for direct RNA sequencing (DRS)

Wild-type or *tent-5(tm3504)* young adult males (50–60 h after hatching at 20 °C) were hand-picked from the male-enriched plates and transferred to an Eppendorf tube containing 10 µl of 50 mM NaCl. After hand-picking males, we did not apply additional washes to avoid sample loss; worms were transferred with minimal bacteria. The worm pellet was then resuspended in 200 µl of TRI Reagent (Sigma-Aldrich), vortexed for 15 min at room temperature, and stored at −80 °C before RNA isolation. This process was repeated three times to obtain sufficient biological material. TRIzol samples from three different pickings were combined just before RNA isolation, resulting in approximately 300 male worms per sample. The wild-type hermaphrodite population was age-synchronized by bleaching gravid adults and seeding isolated embryos on plates. Worms were grown until they reached the young adult stage, then collected and washed three times using 50 mM NaCl. The worm pellet was resuspended in 1 ml of TRI Reagent (Sigma-Aldrich), vortexed for 15 min at room temperature, and stored at −80 °C. RNA was isolated from TRIzol according to the manufacturer's instructions. The samples were additionally purified with KAPA Pure magnetic beads (RNA to beads ratio: 1:3 v/v), and RNA quality was assessed using the Agilent TapeStation system. Samples for wild-type and *tent-5(tm3504)* males and hermaphrodites were prepared in two independent biological replicates.

### Direct RNA sequencing and data analysis

Sequencing libraries were prepared using the DRS Kit (Oxford Nanopore Technologies, SQK-RNA002) according to the manufacturer's protocol. In each library, total RNA isolated from whole male or hermaphrodite worms (0.5 to 1.5 μg, depending on the amount of isolated RNA) was mixed with 2 ng of *in vitro* transcribed poly(A) standards. To monitor tail-length accuracy across runs, we spiked each library with *in vitro*–transcribed RNAs bearing defined poly(A) lengths. Sequencing was performed with the MinION device, followed by basecalling with Guppy 6.0.0 (ONT), mapping to WBCel235 reference (MiniMap v2.17 (Li 2018) with options -k 14 -ax map-ont –secondary = no), and processing with samtools v1.9 (Danecek et al. 2021). The number of reads for each gene was normalized to the total number of reads obtained for each library to account for depth differences. Lengths of poly(A) tails were estimated with Nanopolish (v0.13.2) as described before (Bilska et al. 2020; Liudkovska et al. 2022). Poly(A) length distributions were compared between conditions for transcripts with a minimum of 5 reads per condition using the Wilcoxon test. Adjusted *P*-values were calculated using the Benjamini-Hochberg method. Differential expression analysis was performed using DESeq2 v1.28 Bioconductor package (Love et al. 2014). GO enrichment analysis was performed using WormBase Enrichment Suite (Angeles-Albores et al. 2016). The subcellular localization of proteins was assessed with DeepLoc v2.0 (Thumuluri et al. 2022). DRS data have been deposited to the Gene Expression Omnibus (GEO) database with the accession number GSE271295 and to the European Nucleotide Archive (ENA) with the following accession numbers: ERS20270802, ERS20271308, ERS20270803, ERS20271309, ERS20270804, ERS20270805, ERS20227048, and ERS20270807.

The dataset from Ebbing et al. (2018) for comparisons of sex-enriched genes was reanalyzed from raw *fastq* files deposited by the authors in Gene Expression Omnibus (GEO). Reads were mapped to WBCel235 reference (STAR v2.7.10a (Dobin et al. 2013)) and followed by processing with samtools v1.9 (Danecek et al. 2021). Read counts were collected using featureCounts from the Subread package (v2.0.6 (Liao et al. 2014) with options -Q 10 -p -C -B and WBCel235 annotation). Differential expression analysis was performed using DESeq2 v1.28 Bioconductor package (Love et al. 2014).

For subsequent analyses, groups of genes described in this manuscript were defined using the following filtering criteria. Male-enriched genes are genes significantly enriched in males compared with hermaphrodites (FDR < 0.05). Male-specific genes are genes significantly enriched in males that show no detectable expression in hermaphrodites (FDR < 0.05). The same criteria were applied to hermaphrodite-enriched and hermaphrodite-specific genes. For poly(A) tail length profiling, a transcript was considered elongated if it met the significance threshold (FDR < 0.05) and exhibited an increase in poly(A) tail length of at least 5 nucleotides.

### Spermatids isolation

A day before the spermatids isolation, a few wild-type, *tent-5(tm3504)*, or *tent-5(rtt6)* L4 stage males were transferred to fresh NGM plates seeded with *E. coli* HB101. Separating males from hermaphrodites allows spermatids to accumulate in the male's gonad before the experimental procedure (Singaravelu et al. 2010). Males were dissected the next day in a drop of sperm medium (50 mM HEPES, 50 mM NaCl, 25 mM KCl, 5 mM CaCl_2_, 1 mM MgSO_4_, and 1 mg/ml BSA) by cutting the posterior end of the male with a needle. The isolated spermatids were visualized immediately (Stanfield and Villeneuve 2006; Singaravelu et al. 2010).

### Microscopic observations

Isolated spermatids were imaged using the Olympus FV1000 confocal microscope with a 60x/oil immersion lens. The spermatids’ size was assessed using ImageJ2 software (Rueden et al. 2017) and compared between wild-type and *tent-5(tm3504)* males using a two-tailed unpaired Student's *t*-test. To evaluate the localization of TENT-5 in males, *tent-5::gfp* worms were immobilized with 25 μM levamisole on freshly prepared 2% agarose pads and immediately imaged on the Zeiss LSM800 confocal microscope with a 40×/oil immersion lens or the Olympus FV1000 confocal microscope with a 60x/oil immersion lens.

### Fertility and mating behavior experiments

To assess male fertility, wild-type or *tent-5(tm3504)* L4 stage males were crossed with L4 stage *fog-2(oz40)* females (which are unable to produce their own sperm (Schedl and Kimble 1988)). For each cross, one female and four males were placed on an NGM plate with a 5-mm diameter *E. coli* lawn. Crosses were performed at 20 °C and continued throughout the entire female lifespan. The number of progeny was counted and compared between wild-type and *tent-5(tm3504)* males using a two-tailed unpaired Student's *t*-test. For comparison of mating behavior, wild-type or *tent-5(tm3504)* young adult males were crossed with young adult *unc-45(m94)* hermaphrodites (uncoordinated hermaphrodites were used to increase mating efficiency (Gower et al. 2005)). For each cross, ten hermaphrodites and five males were placed on an NGM plate seeded with *E. coli* HB101 and recorded for 30 min. Recorded movies were subsequently analyzed, and mating behavior was described according to the following criteria: 1—number of different hermaphrodites touched by the male, 2—number of contacts with any hermaphrodite, 3—backward locomotion after touching the hermaphrodite with the tail, 4—turning around the hermaphrodite's body, 5—successful location of the hermaphrodite's vulva, and 6—successful spicule insertion (Gower et al. 2005; Bever et al. 2022). The number of each incident was then compared between wild-type and *tent-5(tm3504)* males using a two-tailed unpaired Student's *t*-test (with or without Welch's correction depending on variance distributions).

## Results

### Male-enriched genes are expressed mainly in sperm, seminal vesicle, and vas deferens

**Fig. 1.**
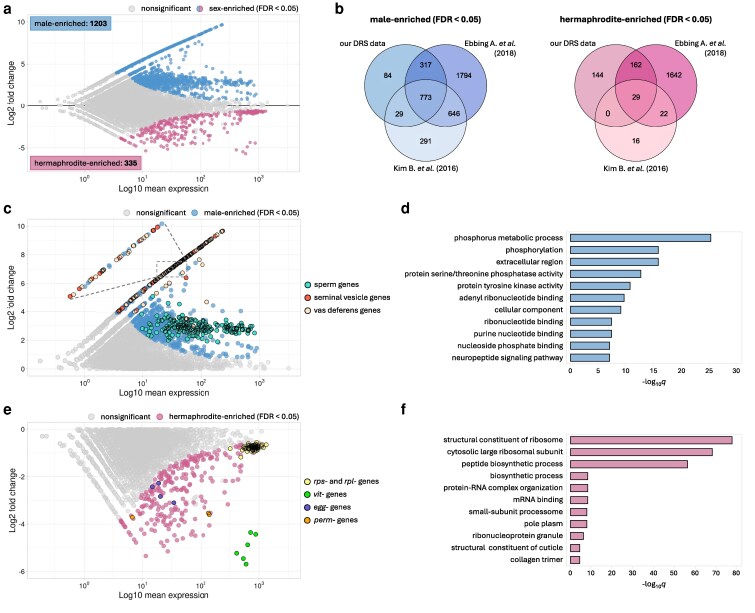
Differences in gene expression patterns between wild-type males and hermaphrodites. a) MA plot illustrating differential gene expression between males and hermaphrodites of N2 wild-type worms. Significantly changed genes (FDR < 0.05) are marked with blue and pink dots for male-enriched and hermaphrodite-enriched genes, respectively. b) Venn diagrams showing overlaps between our DRS data and two other studies comparing transcriptomes of *C. elegans* males and hermaphrodites (Kim et al. 2016; Ebbing et al. 2018). For each compared group, the significantly enriched pool of genes was included according to the cut-off FDR value set in each study. The overlap for male-enriched genes is shown in blue and for hermaphrodite-enriched genes in pink. c) Zoom on the half of the MA plot showing genes upregulated in males. Significantly changed genes (FDR < 0.05) are marked with blue dots. Among these, three of the most interesting groups of genes were selected: sperm genes (cyan dots), seminal vesicle genes (red dots), and vas deferens genes (beige dots). Groups of genes were defined based on Ebbing et al. (2018). A zoomed-in fragment of the plot is shown for clearer visualization of overlapping dots. d) Top GO terms for the male-enriched genes ordered by adjusted *P*-value (WormBase Enrichment Suite). e) Zoom on the half of the MA plot showing genes upregulated in hermaphrodites. Significantly changed genes (FDR < 0.05) are marked with pink dots. Four groups of genes are marked: ribosomal genes (*rpl* and *rps*, yellow dots), vitellogenin genes (*vit*, green dots), *egg* genes (purple dots), and *perm* genes (orange dots). f) Top GO terms for the hermaphrodite-enriched genes ordered by adjusted *P*-value (WormBase Enrichment Suite).

It is widely recognized that gene expression displays significant variability depending on the sex of the organism, contributing to differences in appearance and behavior. Studies of sex-specific gene expression have been conducted in various organisms, including the nematode *C. elegans* (Jiang et al. 2001; Thoemke et al. 2005; Miersch and Döring 2012; Kim et al. 2016; Ebbing et al. 2018). Here, we used Oxford Nanopore Direct RNA Sequencing (DRS) to expand on previous studies. This method allowed us to avoid amplification biases introduced by other PCR-based methods (Brouze et al. 2023) and simultaneously investigate differences in both gene expression and mRNA poly(A) tail length dynamics between young adult *C. elegans* wild-type males and hermaphrodites. Our DRS analysis revealed substantial differences in gene expression between the two sexes, with 1,538 genes exhibiting sex-dependent expression patterns (Fig. 1a, Supplementary Table 1). Among these, 1,203 genes were significantly enriched in males, while 335 were enriched in hermaphrodites. To validate our findings, we compared our results with previously published datasets that utilized different approaches to male collection and sample preparation. For instance, Kim et al. identified 1,739 genes with higher expression in *him-5* mutant males than hermaphrodites using Illumina sequencing (Kim et al. 2016), of which 802 genes overlapped with our study (Fig. 1b, Supplementary Table 1). Similarly, Ebbing et al. detected 3,529 genes upregulated in four individual wild-type males relative to four individual hermaphrodites (Ebbing et al. 2018), with 1,090 genes overlapping with our dataset (Fig. 1b, Supplementary Table 1). The substantial overlap between male-enriched genes across different studies underscores the robustness of our approach and the reliability of our data for elucidating sex-specific gene regulation in *C. elegans*.

We then analyzed the gene sets enriched in males and hermaphrodites in more detail. The Gene Ontology (GO) term analysis of male-enriched genes revealed a distinctive association with phosphatase and kinase activities (Fig. 1c and d), consistent with previous reports identifying these as characteristic features of sperm-related genes (Ma et al. 2014; Kim et al. 2016; West et al. 2018; Cutter et al. 2019). Indeed, among male-enriched genes, we detected a large population encoding sperm components: 30 genes from the *msp* family, 17 from the *ssp* family, and 9 from the *nspd* family (Supplementary Table 1). While both *C. elegans* sexes generate sperm, males exhibit a prolonged sperm production period, resulting in higher overall sperm levels (Singson 2006). Hermaphrodites produce sperm while being in the L4 stage, but here were sequenced as young adults, after the switch to oocyte production. This accounts for the observed upregulation of sperm genes in males compared to hermaphrodites (Fig. 1c). Intriguingly, apart from the sperm genes, many other male-enriched genes are exclusively expressed in males and encode proteins with unknown functions, presenting an opportunity for further exploration in *C. elegans* male research. Subsequent analyses of 297 male-specific genes allowed us to categorize them into two major groups: seminal vesicle genes and vas deferens genes (Fig. 1c). The seminal vesicle and vas deferens are male-specific tissues that are responsible for producing seminal fluid and transporting it into the hermaphrodite uterus during mating (Ebbing et al. 2018). Despite the crucial role of these tissues in reproduction, the precise molecular regulatory mechanisms governing their functions are still not fully understood. However, it is known that due to their secretory function, male-specific tissues are rich in endoplasmic reticulum (ER) (Altun and Hall 2009). In line with that, we observed that 82% of male-specific transcripts (defined as those with no expression in hermaphrodites, FDR < 0.05) are predicted to contain a signal peptide-encoding sequence targeting them to the secretory pathway through the ER (Supplementary Fig. 1, Supplementary Table 1).

The pool of hermaphrodite-enriched genes and their overlap with datasets from other studies appeared relatively small compared to that of males (Fig. 1a and b). However, the genes we identified as enriched closely align with hermaphrodite reproductive functions. As expected, among the genes highly enriched in hermaphrodites (Supplementary Table 1), we found many implicated in hermaphrodite reproduction, germline function, and early embryo development. Notably, all six members of the vitellogenin (*vit*) family exhibited high enrichment in hermaphrodites (Fig. 1e). These genes are predominantly expressed in the intestine of young adult and adult hermaphrodites, encoding yolk proteins responsible for transporting essential nutrients to developing embryos within the gonad (Perez and Lehner 2019 ). Similarly, genes from the *egg* family (e.g. *egg-1*, *egg-2*, *egg-5*, and *egg-6*), *perm* family (e.g. *perm-2*, *perm-4*), and proteins involved in RNA regulation (e.g. *gld-1, lin-41, puf-5, puf-7, puf-11, mex-5, mex-6*), which are all known to influence germ cell proliferation, differentiation, and germline function in hermaphrodites (WormBase), showed substantial enrichment compared to males. Interestingly, we also observed significant differences in the expression of genes encoding ribosomal proteins (*rpl* and *rps*) between the sexes (Fig. 1e and f). Some of these genes (e.g. *rpl-10*, *rpl-3*, *rpl-7A*, *rpl-9, rpl-34, rps-8, rps-11, rps-12, rps-3*) were proposed to play critical roles in germline and embryonic development (WormBase). More broadly, ribosomal proteins are essential for protein synthesis and cell growth. We suspect that their differential expression between sexes might reflect differences in cellular metabolism and growth between hermaphrodites and males.

In conclusion, our DRS results reveal profound differences in gene expression between *C. elegans* sexes, consistent with previous studies (Kim et al. 2016; Ebbing et al. 2018). Our approach provides a more robust basis for examining sex-related transcriptomes by analyzing a large population of wild-type males, thus avoiding the potential influence of the *him* (high incidence of males) mutation background on gene expression and poly(A) tail distributions. Notably, we observed the most pronounced sex-dependent expression for genes encoding poorly studied secreted proteins localized in male-specific tissues.

### Poly(A) tail distributions differ for males and hermaphrodites

**Fig. 2.**
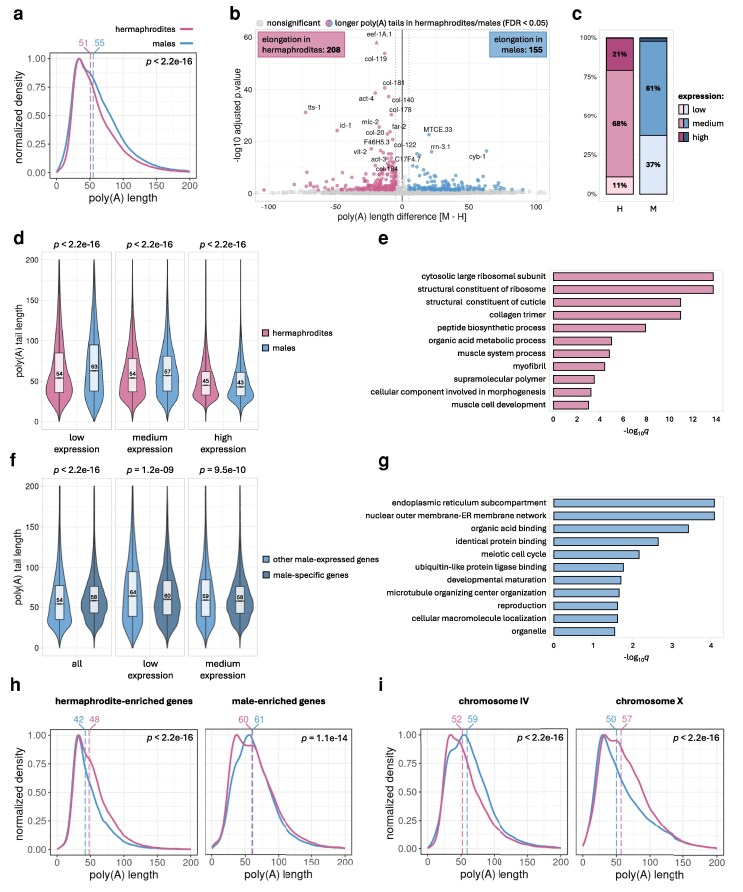
Differences in poly(A) distributions between wild-type males and hermaphrodites. a) Density plot showing global differences in the poly(A) tail distribution between males (blue line) and hermaphrodites (pink line). Vertical dashed lines represent the median poly(A) tail length for each condition (in nucleotides). The plot was generated for all identified transcripts and normalized to 1. b) Volcano plot displaying differential polyadenylation between males and hermaphrodites for N2 wild-type worms. Genes with significantly changed median poly(A) tail length (FDR < 0.05) by a minimum of 5 nucleotides (dotted line) are marked with blue dots (elongated in males) and pink dots (elongated in hermaphrodites). Triangles represent data points outside the *y-*axis limit. “M” in the *x*-axis title represents males and “H” represents hermaphrodites. c) Fractions of genes with elongated poly(A) tails in hermaphrodites (pink bars) or males (blue bars), characterized by low, medium, or high expression levels. Expression levels are defined by baseMean values presented in Supplementary Table 1. Genes with baseMean < 20 are defined as lowly expressed, with 20 < baseMean < 500 as medium expressed, and with baseMean > 500 as highly expressed. “M” in the *x*-axis title represents males and “H” represents hermaphrodites. d) Violin plots showing distributions of poly(A) tail length for genes grouped by their expression levels as defined in panel (c). Numbers inside the boxes indicate the median poly(A) tail length for each condition. e) Top GO terms for genes with poly(A) tails elongated in hermaphrodites ordered by adjusted *P*-value (WormBase Enrichment Suite). f) Violin plots illustrating the distribution of poly(A) tail lengths for male-specific genes compared to other male-expressed genes. Male-specific genes were defined as significantly enriched in males (FDR < 0.05) with no expression in hermaphrodites based on our DRS data (Supplementary Table 1). Other male-expressed genes were defined as all the other genes that were detected in male samples by DRS with minimal baseMean comparable to the lowest expressed male-specific genes (baseMean > 7). The left plot represents the distribution for all male-specific genes contrasted with all others, while the middle and right plots represent similar comparisons within groups of genes with similar expression levels as defined in panel (c). No male-specific genes met our criteria for highly expressed genes. Numbers inside the boxes indicate the median poly(A) tail length for each condition. g) Top GO terms for genes with poly(A) tails elongated in males ordered by adjusted *P*-value (WormBase Enrichment Suite). h) Density plots showing differences in the poly(A) tail distribution for sex-enriched genes between males (blue line) and hermaphrodites (pink line). Vertical dashed lines represent the median poly(A) tail length for each condition (in nucleotides). Plots were generated for all transcripts significantly upregulated in either hermaphrodites or males based on Supplementary Table 1 and normalized to 1. i) Density plots showing differences in the poly(A) tail distribution for all genes localized on chromosome IV or X between males (blue line) and hermaphrodites (pink line). Vertical dashed lines represent the median poly(A) tail length for each condition (in nucleotides). Plots were generated for all transcripts assigned to a particular chromosome and normalized to 1.

Given that the poly(A) tail length often correlates with a transcript's stability and expression level (Brouze et al. 2023), we hypothesized that significant differences would be observed between the two sexes of *C. elegans* not only in gene expression but also in mRNA poly(A) tail length distribution. Utilizing the same DRS-based dataset of mRNAs isolated from hermaphrodites and males as described above, we found global mRNA poly(A) tail length profiles consistent with previous studies (Supplementary Table 2) (Lima et al. 2017; Legnini et al. 2019; Roach et al. 2020; Liudkovska et al. 2022). Additionally, we identified a slight difference between the sexes, with global median poly(A) tail lengths of 51 nt for hermaphrodites and 55 nt for males (Fig. 2a). Further analysis of poly(A) tail length distributions for differentially polyadenylated mRNAs (at least 5 nt difference between sexes, FDR < 0.05) revealed 208 transcripts with significantly longer poly(A) tails in hermaphrodites and 155 transcripts with longer poly(A) tails in males (Fig. 2b, Supplementary Fig. 2a and b, Supplementary Table 2). Interestingly, in males, a significant portion of transcripts with longer tails are lowly expressed (Fig. 2c). This group of mRNAs primarily contributes to the longer median poly(A) tail length observed in males (Fig. 2d). In contrast, many transcripts with longer tails in hermaphrodites than in males encode highly abundant proteins, such as ribosomal proteins, collagens, or actin (Fig. 2c and e, Supplementary Fig. 2c, Supplementary Table 2). These genes were also upregulated in hermaphrodites compared to males based on our differential expression analysis (Fig. 1e and f, Supplementary Table 1). Although within each sex highly expressed genes tend to have shorter poly(A) tails (Fig. 2d), these transcripts display slightly longer tails in hermaphrodites than in males. We speculate that this modest elongation in hermaphrodites may help meet the increased demand for their encoded proteins during embryonic development in the hermaphrodite's gonad.

We observed that in males, poly(A) tail elongation is directed towards transcripts functionally associated with the endoplasmic reticulum (Fig. 2g), suggesting the importance of efficient secretory machinery for proper male physiology. Since most male-specific genes encode proteins secreted by the ER (Supplementary Fig. 1a, Supplementary Table 1), we analyzed their poly(A) tails. We found that globally, male-specific mRNAs exhibit significantly longer poly(A) tails compared to other transcripts expressed in males (Fig. 2f, left panel). This observation can be explained by the relatively higher share of genes with low or medium expression among male-specific genes, which generally have longer poly(A) tails than highly expressed ones (Fig. 2d). Further comparisons of poly(A) tails within groups of genes with similar expression levels revealed lack of consistent and biologically relevant difference in median tail length between male-specific and other male-expressed genes (Fig. 2f, middle and right panels). To investigate further, we also explored the relationship between poly(A) tail lengths and mRNA expression levels. For both hermaphrodites and males, we observed a negative correlation between mRNA tail length and expression level (Fig. 2d and f), which is consistent with previous reports (Lima et al. 2017). Moreover, we found that transcripts significantly upregulated in hermaphrodites also carry globally longer poly(A) tails in hermaphrodites than in males (Fig. 2h). A similar trend was observed for male-enriched transcripts (Fig. 2h).

Finally, due to the distinct chromosome compositions of males and hermaphrodites, we examined the global poly(A) length distributions based on gene location within each chromosome. Surprisingly, we identified differences in poly(A) profiles for transcripts from chromosomes IV and X (Fig. 2i, Supplementary Fig. 2d). Specifically, mRNAs from chromosome IV exhibited significantly longer poly(A) tails in males compared to hermaphrodites, whereas transcripts from chromosome X showed the opposite effect. Although we compared multiple features of genes encoded on each chromosome (expression levels, presence of sequence encoding signal peptides, GC content, transcript length, or 5′ UTR and 3′ UTR length) (Supplementary Fig. 3), we were unable to explain how the observed sex-dependent polyadenylation is influenced by gene chromosome location.

In addition to the significant differences in gene expression between males and hermaphrodites, our DRS results unveil distinct patterns of poly(A) tail length distribution between the two sexes. These findings underscore the important role of poly(A) metabolism in sex-dependent physiological processes.

### TENT-5 regulates the expression of male-specific genes

**Fig. 3.**
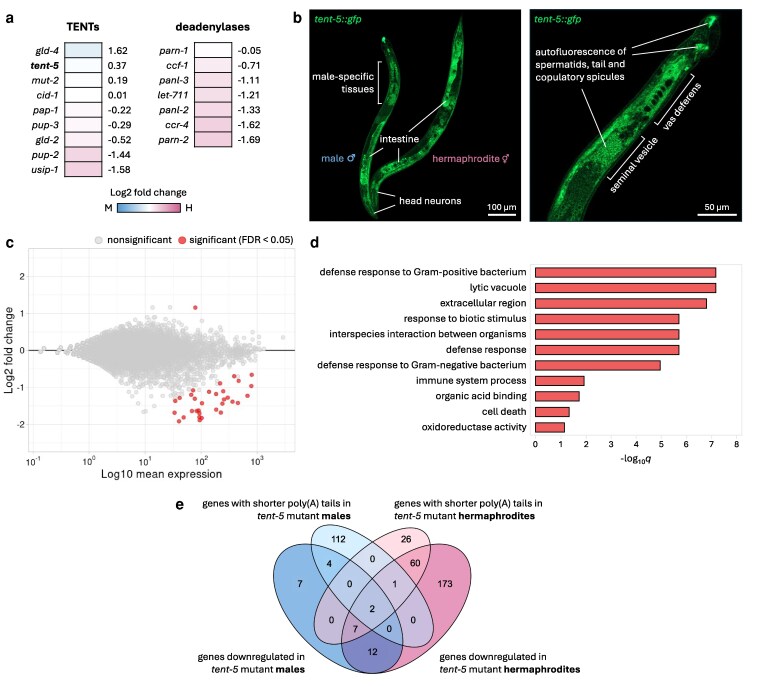
Differences in gene expression patterns between *tent-5* mutant and wild-type males. a) Heatmap showing expression levels of TENTs and deadenylases between males and hermaphrodites. The color of the heatmap specifies whether a gene is upregulated in males (blue) or hermaphrodites (pink). The numbers on the right represent the log2 fold change value derived from Supplementary Table 1. “M” in the heatmap legend represents males and “H” represents hermaphrodites. b) Fluorescence microscopic images of TENT-5-GFP expression in hermaphrodites and males (*tent-5(rtt6[tent-5::gfp::3xflag]* I). The left image represents differences and similarities in TENT-5 expression between sexes. It is composed of six separate shots to show the full body of a male and hermaphrodite. The right image shows the tail-end of a male body. c) MA plot illustrating differential gene expression between wild-type and *tent-5* mutant worms. Significantly changed genes (FDR < 0.05) are marked with red dots. d) Top GO terms for genes significantly downregulated in *tent-5* mutant males ordered by adjusted *P*-value (WormBase Enrichment Suite). e) Venn diagram showing overlaps between our DRS data and our previous study on *tent-5* mutant hermaphrodites (Liudkovska et al. 2022). Four different conditions were compared: genes downregulated in *tent-5* mutant males (blue), genes with shorter poly(A) tails in *tent-5* mutant males (light blue), genes downregulated in *tent-5* mutant hermaphrodites (pink), and genes with shorter poly(A) tails in *tent-5* mutant hermaphrodites (light pink). Included were all significantly downregulated genes (FDR < 0.05) (for differential expression) and all transcripts with significantly shorter poly(A) tail by a minimum of 5 nucleotides (FDR < 0.05) (for differential polyadenylation).

Within the cell, poly(A) tails can undergo modification by various proteins that either lengthen or shorten them (Zhang et al. 2010; Yan 2014; Liudkovska and Dziembowski 2021). We hypothesized that the observed differences in poly(A) distributions between *C. elegans* males and hermaphrodites might result from the sex-specific activities of these proteins. To identify a potential candidate protein, we analyzed our DRS data to determine if the expression of any known nuclear and cytoplasmic poly(A) polymerases and deadenylases varied between the sexes. We observed no significant differences in gene expression levels for any of these poly(A)-modifying enzymes (Fig. 3a, Supplementary Table 1).

Our previous work in hermaphrodite *C. elegans* demonstrated that the polyadenylation of mRNAs encoding ER-targeted secreted proteins is mediated by the cytoplasmic poly(A) polymerase TENT-5 (Liudkovska et al. 2022). Depletion of TENT-5 in hermaphrodites resulted in poly(A) tail shortening and downregulation of mRNAs encoding secreted innate immunity effector proteins, leading to increased susceptibility of *tent-5* mutants to pathogen infection (Liudkovska et al. 2022). As mentioned above, our functional enrichment analysis showed that transcripts with longer poly(A) tails in males compared to hermaphrodites are associated with ER functionality (Fig. 2f). Therefore, we hypothesized that TENT-5 might be responsible for their polyadenylation and thus contribute to the differential polyadenylation observed between the sexes, particularly evident for lowly expressed genes. Additionally, we hypothesized that since most male-specific transcripts are predicted to encode secreted proteins, TENT-5 might also regulate their expression, which could have been overlooked in our previous studies on hermaphrodites.

Using the *tent-5::gfp* knock-in strain, we examined TENT-5 localization in males. Confocal microscopy revealed similar expression of TENT-5-GFP in males and hermaphrodites (Fig. 3b). Notably, we detected a strong TENT-5 signal in male-specific tissues, particularly in the vas deferens (Fig. 3b, Supplementary Fig. 4a), where a large fraction of male-enriched genes is expressed (Fig. 1c). This localization pattern is consistent with the proposed role of TENT-5 in the regulation of male-specific transcripts through polyadenylation. In contrast, TENT-5 expression in seminal vesicle cells could not be reliably assessed based on the available images. Although its presence in these cells cannot be excluded, the current data do not allow a definitive conclusion, and further analysis using an additional transcriptional reporter would be required. In addition, our microscopic observations revealed intense autofluorescence in spermatids as well as at the male tail tip and copulatory spicules (Fig. 3b, Supplementary Fig. 4b and c), a phenomenon previously briefly mentioned in the literature (Yi et al. 2000; Schindelman et al. 2006; Nikonorova et al. 2022; Zuo et al. 2023).

**Fig. 4.**
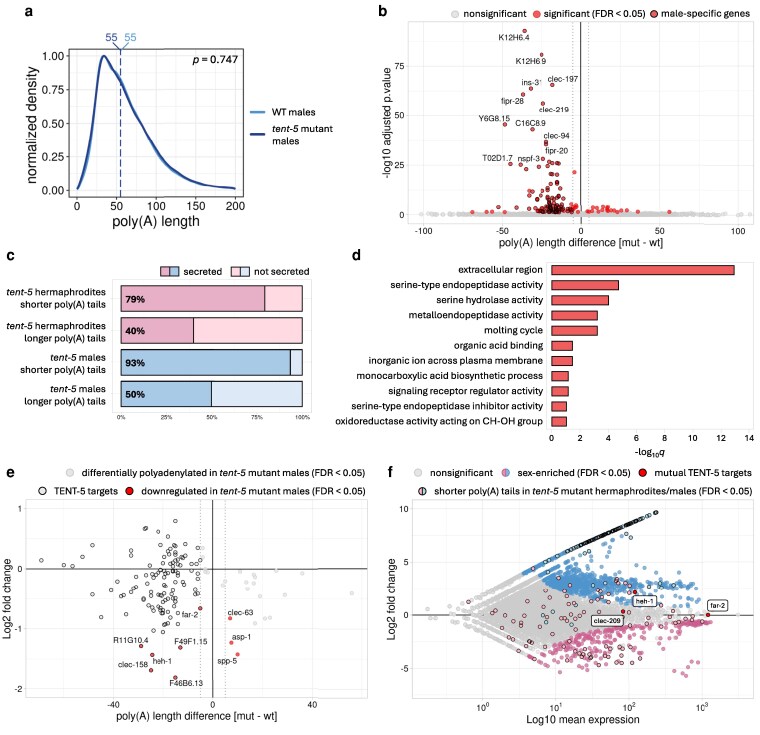
TENT-5 polyadenylates male-specific mRNAs encoding secreted proteins. a) Density plot showing global differences in the poly(A) tail distribution between wild-type (blue) and *tent-5* mutant males (dark blue). Vertical dashed lines represent the median poly(A) tail length for each condition (in nucleotides). The plot was generated for all identified transcripts and normalized to 1. b) Volcano plot showing differential polyadenylation between wild-type and *tent-5* mutant males. Transcripts with significantly changed median poly(A) tail length (FDR < 0.05) are marked with red dots. Black borderline is added to male-specific genes. Male-specific genes were defined as significantly enriched in males (FDR < 0.05) with no expression in hermaphrodites based on our DRS data (Supplementary Table 1). c) Fractions of genes with predicted extracellular localization by DeepLoc 2.0 software (Thumuluri et al. 2022). For each condition, a group of significantly shortened/elongated transcripts by a minimum of 5 nucleotides was included. Data for hermaphrodites comes from Liudkovska et al. 2022. d) Top GO terms for genes with significantly shorter poly(A) tails in *tent-5* mutant males (by a minimum of 5 nucleotides) ordered by adjusted *P*-value (WormBase Enrichment Suite). e) The relationship between the difference in the expression level and the difference in the median poly(A) tail length of respective transcripts for *tent-5* mutant compared to wild-type males. TENT-5 targets (genes with significantly shorter poly(A) tail in *tent-5* mutant worms by a minimum of 5 nucleotides; FDR < 0.05) are highlighted with black borderline. Genes significantly downregulated in *tent-5* mutant males (FDR < 0.05) are marked with red dots. f) MA plot showing differential gene expression between males and hermaphrodites of N2 wild-type worms (Fig. 1a). Significantly changed genes (FDR < 0.05) are marked with blue and pink dots for male-enriched and hermaphrodite-enriched genes, respectively. Additionally, marked are TENT-5 targets (genes with significantly shorter poly(A) tail in *tent-5* mutant worms by a minimum of 5 nucleotides; FDR < 0.05) in males (light blue, black borderline) and hermaphrodites (light pink, black borderline; based on Liudkovska et al. 2022). Red dots with labels represent common TENT-5 targets for both sexes.

To identify TENT-5 substrates in *C. elegans* males and validate the hypothesis that TENT-5 regulates male-specific mRNAs and other transcripts encoding secreted proteins, we performed DRS on *tent-5* mutant and wild-type males, analyzing gene expression and poly(A) patterns. We observed significant dysregulation of 33 genes upon TENT-5 deletion in males: the expression levels of 32 genes were downregulated, and one was upregulated (Fig. 3c, Supplementary Table 3). Notably, the relatively low number of genes with changed expression might be underestimated due to the lower depth of DRS sequencing when compared with commonly used Illumina-based methods. Nevertheless, among the downregulated genes, as many as 6 genes were male-specific, and 17 were male-enriched, which suggests that TENT-5 regulation is indeed relevant for gene expression in males. At the same time, 21 out of 32 downregulated genes were consistent with our previous findings in *tent-5*-deficient hermaphrodites (Fig. 3e, Supplementary Table 3) (Liudkovska et al. 2022), showing similar functional enrichment in defense response processes (Fig. 3d). This underscores TENT-5's role in *C. elegans* innate immunity, independently of nematode sex. However, poly(A) profiling of *tent-5* mutant males revealed even more pronounced sex-related differences. Despite no global change in the poly(A) distribution between wild-type and *tent-5* mutant males (similar to our previous observations for hermaphrodites (Liudkovska et al. 2022)) (Fig. 4a), we detected 137 transcripts with altered poly(A) tail length (changed by at least 5 nt, FDR < 0.05), of which 119 had shorter and 18 longer poly(A) tails in *tent-5* mutant males (Fig. 4b, Supplementary Fig. 5a, Supplementary Table 4). Strikingly, among mRNAs with shortened poly(A) tails in mutants, 94% were either male-specific (100 genes, which accounts for 34% of all male-specific genes described earlier) or male-enriched (12 genes) (Fig. 4b, Supplementary Table 4). As expected, the majority of these transcripts are predicted to encode extracellular proteins (93%), a hallmark of TENT-5 substrates (Mroczek et al. 2017; Bilska et al. 2020; Gewartowska et al. 2021; Liudkovska et al. 2022; Brouze et al. 2024), which is even more pronounced in males than in hermaphrodites (Fig. 4c and d, Supplementary Table 4).

Approximately 66% of TENT-5 targets encode putative components of seminal fluid expressed in male somatic gonad (Supplementary Table 4), suggesting the importance of TENT-5-mediated polyadenylation in regulating certain aspects of male reproduction. For instance, some of these substrates possess serine-type endopeptidase activity (Fig. 4d), which has been previously linked with sperm activation in *C. elegans* (Stanfield and Villeneuve 2006; Smith and Stanfield 2011). Although the process of sperm activation is not well understood in nematodes, it is presumed that inactive spermatids stored in the seminal vesicle are transported through the male's vas deferens and cloaca into the hermaphrodite's uterus. After ejaculation, spermatids are activated by seminal fluid proteins to form functional sperm (Barr et al. 2018). So far, only a few genes involved in this process have been reported (Stanfield and Villeneuve 2006; Smith and Stanfield 2011). Interestingly, one of them (*try-5*) is polyadenylated by TENT-5 based on our results (Supplementary Fig. 5b, Supplementary Table 4).

Among the mRNAs with shortened poly(A) tails in *tent-5* mutant males, we also detected 22 members of the *clec* family (C-type lectins) (Supplementary Fig. 5b, Supplementary Table 4), which are reported to be immune effectors (Pees et al. 2016, 2021). The enrichment of C-type lectins in the seminal fluid suggests their possible function in the nematode's defense response. Recent studies have shown that *C. elegans* males are more resistant to fungal, viral, and bacterial infections, as well as to heat, osmotic, and oxidative stress (Piloto et al. 2022). Also in other species, seminal fluid has been shown to have antimicrobial properties, helping to protect both female and male reproductive tracts against infections (Poiani 2006; Avila et al. 2011). This raises the interesting hypothesis that *C. elegans* males utilize seminal fluid genes for both defense response and mating purposes (Barr et al. 2018). However, the exact mechanism of that possible parallel regulation remains to be established.

Finally, we analyzed how differential gene expression correlates with poly(A) tail length. Although only six genes displayed both significant downregulation and shorter poly(A) tails in mutant males (Figs. 3e and 4e), we observed a trend where TENT-5-mediated poly(A) tail elongation tends to stabilize the corresponding mRNA (Fig. 4e), a pattern also noted in hermaphrodites (Liudkovska et al. 2022 ). This finding suggests that the correlation between mRNA poly(A) tail length and expression level might be largely tissue-specific or specific to certain transcript types or groups. Surprisingly, the overlap between transcripts with shortened poly(A) tails in *tent-5* mutant males and hermaphrodites was minimal (Figs. 3e and 4f), with only three genes (*heh-1*, *far-2,* and *clec-209*) emerging as shared TENT-5 targets across sexes (Supplementary Fig. 5b, Supplementary Table 4). Moreover, *heh-1* and *far-2* were also significantly downregulated in both sexes (Fig. 3e). Although not well studied, these genes are predicted to encode proteins responsible for sterol or fatty acid binding (WormBase). Interestingly, male-specific tissues, where TENT-5 activity in males is most pronounced, are known to be cholesterol-rich (Matyash et al. 2001). Therefore, we speculate that TENT-5 might be involved in cholesterol metabolism in both sexes by stabilizing *heh-1* and *far-2* mRNAs.

Overall, TENT-5 activity in males focuses almost exclusively on polyadenylating male-specific transcripts, whereas in hermaphrodites, it appears much more versatile (Fig. 4f). The functions of most male-specific TENT-5 substrates remain unknown, making it difficult to speculate about TENT-5's physiological role in males. However, it is reasonable to think that the elongation of poly(A) tails of so many putative seminal fluid components in males might be crucial for their proper reproduction.

### TENT-5-mediated poly(A) tail elongation does not affect male mating behavior

**Figure 5.**
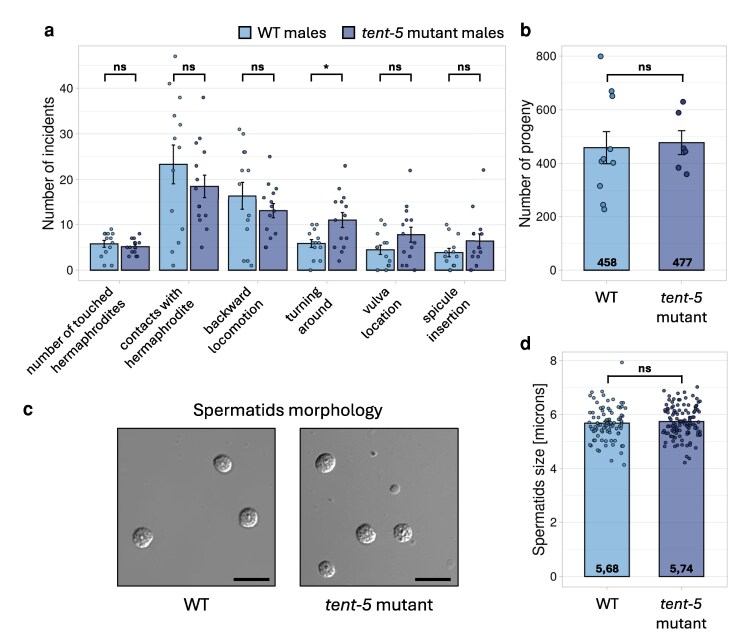
Comparison of mating behavior, fertility, and spermatid morphology between the *tent-5* mutant and wild-type males. (a) Differences between the number of mate-related incidents performed by wild-type (*n* = 15) and *tent-5* mutant (*n* = 14) males. Barplots represent mean values with SD. ns, not significant; *P-value < 0.05 (two-tailed *t-*test). (b) Differences in brood sizes between wild-type (*n* = 10) and *tent-5* mutant (*n* = 6) males crossed with *fog-2* females. Barplots represent mean values with SD. ns, not significant (two-tailed *t-*test). (c) Microscopic DIC images of spermatid morphology for wild-type and *tent-5* mutant males. Scale bars => 10 microns. (d) Differences in spermatid diameter between wild-type (*n* = 86) and *tent-5* mutant (*n* = 114) males. Barplots represent mean values with SD. ns, not significant (two-tailed *t-*test).

Following our observation that TENT-5 is associated with the polyadenylation of male-specific transcripts in *C. elegans* males, we attempted to identify the resulting physiological phenotype. We hypothesized that since TENT-5 targets comprise presumed components of the seminal fluid, the phenotype of *tent-5* mutants might exhibit abnormal mating phenotypes. Firstly, we monitored the mating behaviors of wild-type and *tent-5* mutant males. We did not detect any significant differences in the frequency of male contact with hermaphrodites, characteristic backward movements, vulva searching, or spicule insertion (Fig. 5a, Supplementary Movie 1). The statistically significant difference between wild-type and mutant males was noticed only for the turning behavior. The absence of the obvious mating behavioral phenotype indicates that *tent-5* mutant males have no impaired neuronal or pheromonal signaling. Next, we hypothesized that post-transcriptional changes in male-specific transcripts might influence the potency of the seminal fluid in properly activating male sperm post-ejaculation. To investigate this, we assessed male fertility by counting the offspring produced by *fog-2* females crossed with either wild-type or *tent-5* mutant males. Progeny numbers were comparable between the two conditions (Fig. 5b), which suggests that TENT-5 does not significantly influence male mating behavior or efficiency. These data indicate no detectable effect of TENT-5 loss on male mating behavior or fertility under our conditions. We note, however, that more subtle or environment-dependent phenotypes may require stress, infection, or competition assays to reveal.

Knowing that some seminal fluid components are involved in the process of sperm activation (Stanfield and Villeneuve 2006; Smith and Stanfield 2011), we hypothesized that their TENT-5-mediated regulation might influence the morphology of spermatids stored in the male's seminal vesicle. However, our microscopic observations did not show any alteration in spermatids’ morphology (Fig. 5c, Supplementary Fig. 5c) and size (Fig. 5d). We also did not detect any incidents of mature sperm that were activated before the ejaculation. Nevertheless, considering the strong modifications in seminal fluid components observed at the transcriptome level, we would expect that these changes should manifest as some physiological phenotype that was not examined in this study. More complex *in vivo* studies would need to be performed to elucidate which physiological processes are regulated by TENT-5 poly(A) polymerase in males. Unfortunately, as mentioned above, most of the predicted TENT-5 targets in males do not have any functional annotation, which makes it harder to anticipate their possible function in male physiology.

## Discussion

Advancements in methods that allow precise genome-wide estimation of the poly(A) tail length and composition have significantly boosted our understanding of poly(A) tail metabolism and its role in gene regulation (Brouze et al. 2023). However, it quickly became evident that this regulation is highly cell- and tissue-type specific, and extremely dynamic in response to environmental and developmental cues. Therefore, it should be explored in comprehensive ways that can capture these dynamic changes. Several excellent studies leveraged these novel methodologies to profile poly(A) tail length during oocyte maturation in *Drosophila melanogaster* (Lim et al. 2016) and *Xenopus laevis* (Yang et al. 2020), mammalian oocyte-to-embryo transition (Xiong et al. 2022), and embryonic development of *Danio rerio*, *X. laevis*, and *C. elegans* (Subtelny et al. 2014; Legnini et al. 2019; Roach et al. 2020). However, sex-related differences in global poly(A) metabolism remain largely overlooked in most organisms. In this study, we employed Direct RNA Sequencing to investigate poly(A) tail distribution differences between hermaphrodite and male *C. elegans*, aiming to understand their underlying reasons and consequences.

We observed clear, previously reported differences in gene expression between males and hermaphrodites, reflecting the distinct cellular metabolism, growth, and physiology of adult hermaphrodites and males. Additionally, our poly(A) profiling revealed a distinct sex-dependent distribution of poly(A) tail length. In general, our data align with previous observations that highly abundant housekeeping genes possess relatively short poly(A) tails (Castellano and Bazzini 2017; Lima et al. 2017). We confirmed this negative correlation between mRNA poly(A) tail length and expression level for *C. elegans* males and hermaphrodites. Notably, highly expressed mRNAs have slightly shorter tails in males compared to hermaphrodites, whereas lowly expressed mRNAs have significantly longer tails in males. Interestingly, sex-enriched mRNAs (those expressed in both sexes but upregulated in one) tend to have longer tails in the respective sex. The mechanism behind this sex-dependent difference remains unclear. We also noted chromosomal differences in poly(A) distributions. Transcripts encoded by genes on the X chromosome have longer poly(A) tails in hermaphrodites, while those derived from chromosome IV have longer tails in males. The X chromosome is enriched with female transcripts and largely devoid of male-enriched ones (Albritton et al. 2014; Cutter et al. 2019), likely leading to the observed global elongation of poly(A) tails for X chromosome transcripts in hermaphrodites. Conversely, the opposite trend for chromosome IV can be explained by the presence of many male-enriched, spermatogenesis-related genes (Miller et al. 2004; Wang and Reinke 2008). Another notable observation concerns male-specific transcripts. We found that 82% of these transcripts are predicted to contain signal peptide-encoding sequences targeting them to the secretory pathway through the ER. Interestingly, this characteristic resembles the GO term enrichment observed for genes with poly(A) tails elongated in males compared to hermaphrodites, that similarly pointed out to ER-related genes. However, when compared with other mRNAs expressed in males, male-specific transcripts show no consistent increase of their median poly(A) tail length.

The mechanistic reasons for the sex-specific differences in poly(A) tail distributions are not yet fully understood. Multiple factors influence mRNA poly(A) tail length, with the enzymatic activities of deadenylases, noncanonical poly(A) polymerases, and terminal uridylyltransferases playing primary roles (Zhang et al. 2010; Yan 2014; Liudkovska and Dziembowski 2021). The steady-state poly(A) distribution at the transcriptome scale is achieved through the cell- or tissue-specific concentrations of these enzymes and their varying affinities modulated by auxiliary factors toward particular transcripts. We discovered that for a specific group of mRNAs, which predominantly encode secreted components of seminal fluid, the poly(A) tail length is affected by the TENT-5 ncPAP. TENT-5 localizes to the ER-rich vas deferens, consistent with stabilization of male-specific secretory mRNAs. In hermaphrodites, the same enzyme is mainly expressed in the intestine and enhances the expression of genes encoding secreted innate immune effectors (Liudkovska et al. 2022). Therefore, in both *C. elegans* sexes, TENT-5 polyadenylates transcripts encoding proteins with predicted extracellular localization, underscoring the notion that the TENT5 family of ncPAPs is essential for post-transcriptional regulation of mRNAs encoding proteins directed towards the ER secretory pathway (Bilska et al. 2020; Gewartowska et al. 2021; Liudkovska et al. 2022; Brouze et al. 2024). As described in our previous work (Liudkovska et al. 2022), the exact mechanism behind TENT-5-substrate specificity is not yet clear. However, as a fraction of TENT-5 resides in the ER membrane, we proposed that TENT-5 specificity is directly or indirectly driven by its colocalization with ER-targeted mRNAs (Liudkovska et al. 2022). We have previously predicted that cytoplasmic polyadenylation by TENT-5 would be most pronounced in tissues with high secretion capacity. Consequently, in males, it is not surprising that TENT-5 focuses on regulating male-specific genes expressed primarily in the ER-rich vas deferens. Furthermore, our data strongly suggest that TENT-5 affects the composition of seminal fluid by modulating its transcriptome, potentially playing a role in male reproduction. However, despite intensive efforts, we have not identified phenotypes related to fertility or mating behavior in TENT-5-deficient males, possibly because these phenotypes manifest only under specific environmental conditions. We also cannot exclude the possibility that other ncPAPs may redundantly function with TENT-5 specifically in male tissues.

From an evolutionary perspective, it is interesting to examine the role of noncanonical cytoplasmic poly(A) polymerases in reproduction and gametogenesis across different animal species. In *C. elegans*, GLD-2 is a crucial regulator of the germline mitosis-to-meiosis transition and subsequent gametogenesis (Nousch et al. 2017). Consequently, GLD-2 depletion in nematodes results in defective oocytes and sperm production, leading to infertility in both hermaphrodites and males (Boag et al. 2018). Similarly, two homologs of GLD-2 in *D. melanogaster*, Gld2 and Wispy, are essential for proper gamete production in a sex-dependent manner (Benoit et al. 2008; Sartain et al. 2011). Gld2 is expressed exclusively in the male germline and is required for the completion of spermatogenesis; its absence results in complete sterility in male flies (Sartain et al. 2011). Wispy, on the other hand, regulates late oogenesis and is crucial for female fly fertility (Benoit et al. 2008). Surprisingly, the knockout of the mouse homolog of GLD-2, TENT2, does not cause abnormalities in gamete production or affect reproduction (Nakanishi et al. 2007). In mammals, the TENT5 family of ncPAPs, which includes four members (TENT5A-D), plays an essential role in spermatogenesis and oogenesis (Brouze et al. 2024). In mice, TENT5C and TENT5D are expressed in spermatocytes and spermatids, regulating different aspects of spermiogenesis (Zheng et al. 2019; Cong et al. 2022; Brouze et al. 2024). Depletion of either TENT5C or TENT5D results in complete male infertility (Brouze et al. 2024). Additionally, TENT5C and TENT5B regulate oogenesis, and the double knockout mutation of both proteins leads to infertility (Brouze et al. 2024). In contrast, TENT-5 poly(A) polymerase in worms is not essential for hermaphrodite fertility (Liudkovska et al. 2022) and only slightly impacts the male reproductive system. These findings indicate that the role of cytoplasmic polyadenylation is highly conserved across species. However, different species may rely on the activities of different TENTs to varying extents.

Further research is needed to uncover the complexity of poly(A) tail metabolism at the organismal level, not only in gametogenesis and reproduction but also in many other physiological processes. Our DRS analysis represents an important step toward expanding the current understanding of cytoplasmic polyadenylation. Additionally, we believe our results provide a foundation for further studies on *C. elegans* male physiology.

## Supplementary Material

jkag058_Supplementary_Data

## Data Availability

The Nanopore Direct RNA Sequencing data have been deposited to the Gene Expression Omnibus (GEO) database with the accession number GSE271295 and to the European Nucleotide Archive (ENA) with the following accession numbers: ERS20270802, ERS20271308, ERS20270803, ERS20271309, ERS20270804, ERS20270805, ERS20227048, and ERS20270807. Supplemental material available at G3 online.
