# Supplementary material for: TENT-5 polyadenylates and regulates male-specific transcripts in Caenorhabditis elegans
Source: G3 (Bethesda). 2026 Mar 23;16(5):jkag058. doi: 10.1093/g3journal/jkag058 (PMC13148395; doi:10.1093/g3journal/jkag058)
Supplement: jkag058_Supplementary_Data [file jkag058_supplementary_data.zip › Reference.docx]

**Reference:**

Suh J, Hutter H. 2012. A survey of putative secreted and transmembrane proteins encoded in the C. elegans genome. BMC Genomics 13:333. https://doi.org/10.1186/1471-2164-13-333.
